# Influence of diet on the modulation of gut microbiota and its neurobiological effects on cognition: a systematic review and meta-analysis

**DOI:** 10.29219/fnr.v70.13843

**Published:** 2026-06-16

**Authors:** Alexander Pabón Moreno, Edgar Fabián Manrique-Hernández, Maricel Licht-Ardila, Alexandra Hurtado-Ortiz, Melissa Melinna Gómez Arrieta, Rhonald Gómez Caballero, Sol Ochoa, Diana Carolina Tiga Loza, Silvana Teresa Tapia Paniagua, Hernan Guillermo Hernández Hincapié

**Affiliations:** 1 Postgraduate Department in Infectious Disease, Universidad de Santander, Bucaramanga, Colombia; 2 Hospital Internacional de Colombia HIC-Fundación Cardiovascular de Colombia FCV- Fundación Universitaria FCV, Vereda de Menzuly, Piedecuesta, Colombia; 3 Escuela de Graduados, Universidad Nacional de Córdoba, Córdoba, Argentina; 4 Universidad de la Sabana, Cundinamarca, Colombia; 5 Departamento de Microbiología, Universidad de Málaga, Málaga, España; 6 Health Sciences Division, Faculty of Dentistry, Comprehensive Oral Health Research Group - SIB, UNiversidad Santo Tomás, Bucaramanga, Colombia

**Keywords:** dietary patterns, gastrointestinal microbiomes, cognition disorders, mild cognitive impairment, brain–gut axis, neurodegenerative diseases

## Abstract

**Introduction:**

The gut microbiota, a key regulator of the gut-brain axis, is profoundly influenced by diet, and its modulation through dietary patterns may play a critical role in mitigating mild cognitive impairment (MCI).

**Objective:**

To evaluate whether dietary interventions modify gut microbiota composition in individuals with MCI and to determine whether these microbiota changes are associated with variations in cognitive function.

**Methods:**

A systematic review and meta-analysis were conducted in accordance with Preferred Reporting Items for Systematic Reviews and Meta-Analysis guidelines. Randomized clinical trials and observational studies evaluating dietary exposures, gut microbiota, and cognitive outcomes in adults with MCI were included. Data extraction and risk-of-bias assessment were performed using standardized and validated tools. When applicable, pooled effects on cognition were estimated using random-effects models and between-study heterogeneity was quantified.

**Results:**

Of 3,029 records identified, 11 studies met the inclusion criteria (nine randomized trials and two observational studies), with a weighted mean age of 65.24 years. Gut microbiota differed between normal cognition and MCI-beneficial taxa (e.g. *Bifidobacterium, Faecalibacterium*) predominated in normal cognition, while potentially taxa more frequently reported in pro-inflammatory/dysbiosis-associated profiles (e.g. *Ruminococcus, Enterobacteriaceae*) were higher in MCI. The pooled effect showed a trend favoring dietary interventions (standardized mean difference = −0.32 (95% CI: −0.92 to 0.28)), although this did not reach statistical significance.

**Conclusion:**

This review highlights the potential role of diet in modulating gut microbiota and its impact on cognition in MCI, emphasizing the need for standardized interventions and further research to clarify the underlying mechanisms.

## Popular scientific summary

- This systematic review and meta-analysis examines the association between dietary patterns, gut microbiota composition, and cognitive outcomes in individuals with mild cognitive impairment (MCI), a key stage preceding dementia.
- By integrating evidence from clinical trials and observational studies, we provide a pooled estimate of the effect of diet-based interventions on cognition and a concise, taxa-level summary of microbiota changes linked to dietary exposure.
- Despite heterogeneity and generally small sample sizes, the available evidence supports diet as a feasible non-pharmacological approach. Larger, standardized studies are needed to clarify diet–microbiota–cognition pathways and their potential role in slowing cognitive decline.

The gut microbiota is a complex community of microorganisms in the human gastrointestinal tract that plays a crucial role in neurological health and is profoundly influenced by diet (1–3). Alterations in its composition and functionality can contribute to the development of conditions such as mild cognitive impairment (MCI) (4). This microbial ecosystem is not only responsible for digestion and nutrient absorption but also regulates the immune system, metabolism, and the gut–brain axis, a bidirectional communication pathway between the gut and the brain (5, 6). Recent research has shown that changes in the gut microbiota can trigger and exacerbate various disorders, highlighting its importance in the prevention and management of MCI (7–9).

MCI is characterized by a noticeable decline in cognitive abilities that does not significantly interfere with daily activities and is a critical stage between normal cognitive aging and dementia (10). This condition can be a precursor to more severe neurological diseases, such as Alzheimer’s disease and other dementias (11, 12). Therefore, identifying factors that modulate the progression and manifestation of MCI is crucial. Understanding these factors is essential for developing effective preventive measures and therapeutic strategies aimed at slowing the progression or potentially reversing the effects of cognitive decline associated with MCI.

Diet emerges as a key modulatory factor that can significantly influence the composition and functionality of the gut microbiota (13). The Western diet, rich in saturated fats, sugars, and processed foods, is associated with reduced microbial diversity and increased intestinal permeability (14, 15). These changes can contribute to systemic inflammation and neuroinflammation, factors implicated in cognitive decline and the development of neurological diseases (16, 17). In contrast, dietary patterns rich in fibers, polyphenols, and omega-3 fatty acids, such as the Mediterranean diet, have shown beneficial effects in modulating the gut microbiota, promoting greater microbial diversity and the production of anti-inflammatory metabolites (18).

However, despite increasing recognition of the gut–brain axis as a mediator between diet and cognition, the evidence specific to MCI remains fragmented and inconsistent. Current trials and observational studies employ heterogeneous dietary exposures, microbiome profiling techniques, and cognitive assessment tools, which hinder cross-study comparability and synthesis (19). Few investigations have simultaneously assessed diet, microbiota, and cognition to determine whether microbial shifts accompany or mediate cognitive effects. Moreover, metabolomic integration, essential for linking microbial metabolites with neurobiological outcomes, remains scarce.

Accordingly, the present review uniquely consolidates MCI-specific human evidence by pairing a quantitative synthesis of cognitive outcomes with a structured synopsis of diet-associated microbiota signals reported across studies. Notably, the few interventional studies exploring integrative dietary approaches in MCI are small-scale and mechanistic, such as the modified Mediterranean–ketogenic diet trial demonstrating associations between gut microbial changes, short-chain fatty acids (SCFAs), and Alzheimer’s disease biomarkers in cerebrospinal fluid (20). This fragmentation limits the ability to quantify the magnitude and direction of dietary effects on cognition in MCI and underscores the need for comprehensive, standardized, and mechanistically oriented research.

The objective of this study was to analyze the relationship between specific dietary patterns, changes in gut microbiota composition, and cognitive outcomes in adults with MCI. Through a systematic review and meta-analysis, the study quantified associations between diet and cognition and summarized microbiota features reported alongside dietary exposures, with particular attention to whether observed microbiota modulation accompanied the relationship between diet and cognition. The findings consolidate current evidence and inform priorities for future mechanistic and interventional research and for clinically oriented nutrition guidance.

## Methods

A systematic review and meta-analysis were conducted following the Preferred Reporting Items for Systematic Reviews and Meta-Analysis (PRISMA) guidelines (21). The study protocol was prospectively registered in the PROSPERO database to ensure transparency and reproducibility (CRD42024540754) (22).

### Eligibility criteria (PICOS)

*Population (P):* We included studies enrolling adults (registered in tMCI, defined by recognized diagnostic criteria or objective cognitive testing with preserved functional independence. Studies including cognitively normal (CN) participants were eligible when CN data were used as comparators or when MCI-specific results could be extracted.

*Intervention/Exposure (I):* Eligible dietary exposures or interventions included whole dietary patterns or modifications (e.g. Mediterranean-type diets, ketogenic variants, fiber- or polyphenol-rich diets) and/or defined dietary components or supplements (e.g. probiotics or prebiotics), provided that the dietary exposure was clearly described or assessed.

*Comparison (C):* Comparators included CN participants, placebo or usual diet, alternative dietary interventions, or exposure strata, as appropriate for the study design.

*Outcomes (O):* Studies were required to report at least one cognitive outcome assessed using validated instruments and/or MCI diagnostic status. Gut microbiota outcomes – including composition, diversity indices, targeted taxa, or microbiota-derived metabolites – assessed using culture-independent methods (e.g. 16S Ribosomal Ribonucleic Acid sequencing, metagenomics, Quantitative Polymerase Chain Reaction) were extracted when available.

*Study design (S):* Eligible designs included randomized or non-randomized clinical trials and observational studies (cohort, case–control, or cross-sectional) published as full-text articles. We included studies published in any language up to 2025. We excluded animal or in vitro studies, case reports or case series, narrative or systematic reviews, conference abstracts without sufficient data, studies exclusively involving established dementia or Alzheimer’s disease without separable MCI data, and studies lacking a clearly defined dietary exposure or intervention.

For transparency, studies were synthesized according to the outcomes they reported: cognitive outcomes were included in the quantitative meta-analysis, whereas microbiota outcomes were summarized qualitatively at the taxa level.

#### Search strategy and selection process

The literature search was conducted initially in June 2024 and updated in October 2025 by two independent reviewers, without restrictions on language or publication date. Electronic databases searched included PubMed, MEDLINE, EMBASE, Scopus, LILACS, SciELO, SAGE, Springer, and the Cochrane Library. Search strategies combined Medical Subject Headings (MeSH) and free-text terms related to cognition, gut microbiota, the gut–brain axis, and diet using Boolean operators (AND/OR). Grey literature sources, including Google Scholar and academic thesis repositories, were also screened. A snowballing approach was subsequently applied to identify additional eligible studies from reference lists. The detailed search strategies for each database are presented in Table 1.

**Table 1:** Search terms for the meta-analysis

Database	Search terms
ScienceDirect	(‘Cognitive Dysfunction’ OR ‘Cognition Disorders’ AND (‘Gastrointestinal Microbiome’ OR Microbiota OR ‘Brain-Gut Axis’) AND (Diet OR ‘Healthy Diet’)
PubMed	(‘Cognitive Dysfunction’ OR ‘Cognition Disorders’ OR Cognition) AND (‘Gastrointestinal Microbiome’ OR Microbiota OR ‘Brain-Gut Axis’) AND (Diet OR ‘Healthy Diet’)
Embase	(‘Cognitive Dysfunction’/exp OR ‘Cognition Disorders’/exp OR Cognition) AND (‘Gastrointestinal Microbiome’/exp OR Microbiota OR ‘Brain-Gut Axis’/exp) AND (Diet OR ‘Healthy Diet’)
SAGE Journals	(‘Cognitive Dysfunction’ OR ‘Cognition Disorders’ OR Cognition) AND (‘Gastrointestinal Microbiome’ OR Microbiota OR ‘Brain-Gut Axis’) AND (Diet OR ‘Healthy Diet’)
Springer	(‘Cognitive Dysfunction’ OR ‘Cognition Disorders’ OR Cognition) AND (‘Gastrointestinal Microbiome’) AND (Diet)
Scopus	(‘Cognitive Dysfunction’ OR ‘Cognition Disorders’ OR Cognition) AND (‘Gastrointestinal Microbiome’ OR Microbiota OR ‘Brain-Gut Axis’) AND (Diet OR ‘Healthy Diet’)

#### Process of selection

Study screening was conducted in Rayyan (23) using a blinded and independent approach. Two reviewer pairs (AH/MG and RG/AP) independently assessed records and classified each article as eligible or ineligible. Screening was performed in two stages: initial title and abstract review, followed by full-text assessment of potentially relevant studies. Discrepancies were resolved through discussion and consensus; when agreement could not be reached, a third reviewer adjudicated.

#### Data collection process

Data were collected, such as authors’ names, year, title, country, study design, sample size, gender classification, age, and standard deviation. Main characteristics of the diet type, microbiome, changes in microbiome composition, and cognitive status were recorded in a data extraction form. A standardized data extraction form was developed, piloted, and refined prior to full data extraction. Information on funding sources and conflicts of interest was also collected to assess potential reporting bias.

#### Assessment of risk of bias in studies

Risk of bias was assessed with the Newcastle–Ottawa Scale (NOS) for observational studies, and star counts were tabulated in three domains (selection, comparability, and outcome/exposure). Studies were classified as high quality (9–10 points), acceptable quality (6–8 points), and low quality (<6 points). For clinical trials, risk of bias was evaluated using the Cochrane Risk of Bias 2.0 (RoB 2) tool, where studies were classified as low risk, some concerns, or high risk of bias, according to the criteria established for randomized trials. The Grading of Recommendations Assessment, Development and Evaluation (GRADE) method was used to assess the evidence level. Two independent reviewers conducted the risk of bias assessment for each study. Any discrepancies in assessments were resolved through discussion, and if necessary, a third reviewer was consulted to reach consensus.

#### Effect measures

Various effect measures were used to synthesize and present data for each of the outcomes analyzed in this systematic review and meta-analysis. For diet-related outcomes concerning cognitive status, combined means and standard deviations were calculated to represent the distribution and variability of these parameters in the studied population. Standardized Mean Difference (SMD) was used to compare diet types between groups with cognitive impairment, allowing assessment of the magnitude of the diet’s effect on cognition.

#### Synthesis methods

A rigorous selection process was used to determine eligibility for each synthesis, including tabulating characteristics of dietary interventions. Data were collected on the type of diet (e.g. Mediterranean diet, high-fiber diets, probiotic supplementation), which were then grouped as follows: Diets based on specific foods: (Flavanols (diet rich in flavanols / flavanol-based dietary intervention), diet rich in methylxanthine), traditional and modified diets: (Mediterranean, Modified Mediterranean–Ketogenic Diet), supplementation and specific components: (probiotics, 1,3-Dimethyluric acid, paraxanthine), general health diet: (healthy diet).

Data on the target population (e.g. age, health status), intervention duration, and outcomes measured in both gut microbiota and MCI were also identified. These characteristics were compared against predefined inclusion and exclusion criteria to ensure that only relevant studies were included in the synthesis.

To prepare the data, we addressed calculating the mean difference along with its standard deviation. Summary tables were created detailing key characteristics and results of each study, enabling clear and direct comparison. Additionally, forest plots were used to visualize individual study results and overall synthesis, aiding in the interpretation of dietary interventions’ effects on MCI. Flowcharts were also employed to illustrate the study selection process and reasons for exclusion. For the meta-analysis, random-effects models were applied due to the observed heterogeneity among the studies. Heterogeneity was assessed using the I^2^ statistic and Cochran’s Q test. Studies were excluded from the meta-analysis if outcomes were not reported in a poolable format, if endpoints were not comparable, or if essential data were missing. Software packages such as Stata/MP version 16.0 (StataCorp, College Station, TX, USA) and R version 4.4.0 were utilized for conducting these analyses.

## Results

**Fig. 1:**
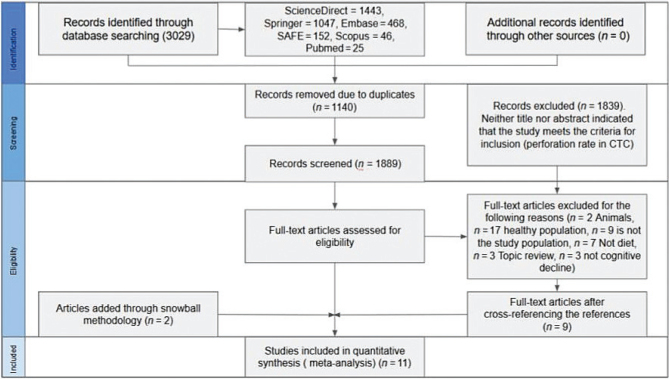
Preferred Reporting Items for Systematic Reviews and Meta-Analysis flow chart of articles search methods and selection.

The search conducted across different databases yielded a total of 3,029 studies, from which duplicates were removed, leaving 1,889 unique articles. These were then subjected to an initial review, resulting in the exclusion of 1,839 articles for not meeting inclusion criteria, excluded at title/abstract screening. Fifty full-text articles were evaluated, out of which 41 were excluded for various reasons: studies involving animals (2), studies involving healthy populations (17), studies not focusing on the target population (9), studies not addressing diet (7), and review articles (3), as well as those not involving cognitive impairment (3). Two additional articles were identified through snowball sampling. Ultimately, 11 studies met the inclusion criteria for the systematic review and meta-analysis (Fig. 1).

Among the 11 included studies, five were conducted in the United States (45.45%) and two in China (18.18%). One study each originated from France and Japan (9.09% each), one was reported as conducted across the United Kingdom and Australia (9.09%), and one was multicenter (9.09%). Nine studies were randomized controlled trials and two were observational studies (one prospective cohort and one nested case–control).

The included studies were published between 2018 and 2023, with 2023 accounting for the largest proportion (27.27%). Overall, 4,351 participants were included across all studies. The largest sample was reported by Zhang et al. (*n* = 2,239), followed by González-Domínguez et al. (*n* = 842). The weighted mean age was 65.24 years (SD 7.33). Regarding the type of cognitive impairment, 54.54% of studies reported having a population with both no impairment and MCI, 27.27% reported only MCI, and 18.18% reported no cognitive impairment.

Risk of bias was assessed using the NOS for observational studies and RoB 2 for randomized trials. Observational studies demonstrated overall good methodological quality on the NOS (Table 2). For randomized trials, RoB 2 judgments ranged from low risk to high risk across domains. Using GRADE, the certainty of evidence for trial outcomes was rated as moderate overall; two trials were judged at high risk of bias, driven primarily by missing outcome data, and one of these also presented concerns regarding selection of the reported result (Tables 2 and 3).

**Table 2:** Bias assessment and level of evidence score Newcastle–Ottawa

Case and control study	Selection				Comparability	Outcomes			GRADE
	Is the case definition adequate	Representativeness of the cases	Selection of controls	Definition of controls	Comparability	Ascertainment of exposure	Same method of ascertainment for cases and controls	Non-response rate	
González-Domínguez et al., 2021	⋆	⋆	⋆	⋆	⋆⋆	⋆	⋆	⋆	High ꚚꚚꚚꚚ
Cohort study	Representativeness of the exposed cohort	Selection of the non-exposed cohort	Ascertainment of exposure	Demonstration that the outcome of interest was not present at the start of the study	Comparability of cohorts on the basis of the design or analysis	Assessment of the outcome	Was follow-up long enough for outcomes to occur	Adequacy of follow up of cohorts	
Zhang et al., 2021	⋆	⋆	⋆	⋆	⋆⋆	⋆	⋆	⋆	Moderate ꚚꚚꚚO

NOS: Newcastle–Ottawa Scale; GRADE: Grading of Recommendations Assessment, Development and Evaluation. Stars indicate risk-of-bias/quality items met within each domain (Selection, Comparability, Outcome/Exposure), following the NOS guidance for the corresponding study design (case–control/cohort/cross-sectional). A higher number of stars indicates lower risk of bias. For comparability, up to two stars can be awarded. Overall quality categories were assigned based on the total star count as reported in the Methods, and the GRADE certainty label reflects the overall confidence in the evidence for the study set/endpoint considered.

**Table 3:** Bias assessment and level of evidence score Cochrane Risk of Bias Tool

Study	D1. Bias arising from the randomization process	D2. Bias due to deviations from intended interventions	D3. Bias due to missing outcome data	D4. Bias in measurement of the outcome	D5. Bias in selection of the reported result	Overall risk of bias	GRADE
Vauzour et al., 2023 (CANN trial; results paper)	Low	Low	Some concerns	Low	Some concerns	Some concerns	Moderate ꚚꚚꚚO
Nagpal et al., 2019 (20)	Some concerns	Low	Some concerns	Low	Some concerns	Some concerns	Moderate ꚚꚚꚚO
Nagpal et al., 2020 (24)	Some concerns	Low	Some concerns	Low	Some concerns	Some concerns	Moderate ꚚꚚꚚO
Dilmore et al., 2023	Some concerns	Low	Some concerns	Low	Some concerns	Some concerns	Moderate ꚚꚚꚚO
Sakurai et al., 2022	Low	Low	Low	Low	Low	Low	Moderate ꚚꚚꚚO
Mashael et al., 2022	Low	Low	Some concerns	Low	Some concerns	Some concerns	Moderate ꚚꚚꚚO
Yuzhe et al., 2023	Some concerns	Low	Low	Some concerns	Some concerns	Some concerns	Moderate ꚚꚚꚚO
McLeod et al., 2023	Some concerns	Low	High	Low	High	High	Moderate ꚚꚚꚚO
Ghosh et al., 2020 (25)	Some concerns	Some concerns	High	Low	Low	High	Moderate ꚚꚚꚚO

RoB 2: Cochrane Risk of Bias tool for randomized trials; GRADE: Grading of Recommendations Assessment, Development and Evaluation. Domains: D1 (randomization process), D2 (deviations from intended interventions), D3 (missing outcome data), D4 (measurement of the outcome), D5 (selection of the reported result). Judgments are Low risk, Some concerns, or High risk, and the overall risk-of-bias reflects the highest level of concern across domains according to RoB 2 guidance. The GRADE certainty label summarizes the overall confidence in the evidence for the trial outcomes included in the synthesis and was informed by risk of bias, inconsistency, indirectness, imprecision, and publication bias.

To facilitate interpretation, study findings were synthesized by design and outcome domain (Table 4). Across clinical trials included in the quantitative synthesis, dietary strategies comprised Mediterranean-style patterns (with or without weight-loss components), probiotic supplementation, and targeted nutritional components. Across studies, microbiome outcomes were heterogeneous: some trials reported shifts in specific taxa and/or alpha/beta diversity, whereas others reported minimal changes. Cognitive findings were similarly variable across domains and instruments, consistent with the pooled estimate and the observed between-study heterogeneity reported below.

**Table 4:** Characteristics and main findings of randomized controlled trials and observational studies evaluating dietary factors and probiotic interventions in relation to gut microbiota (or microbiota-derived markers) and cognitive outcomes in older adults with MCI or cognitive concerns

Study	Diet type	Intervention (vs comparator)	Duration / design	Sample size	Main outcomes	Main findings (high level)
Vauzour et al., 2023 (CANN)	Dietary supplementation (*ω*-3 + cocoa flavanols)	OM3FLAV (EPA/DHA + cocoa flavan-3-ols) vs control	Randomized, controlled, parallel design; 12 months; double-blind	Randomized *n* = 258; baseline measures *n* = 246 final measures *n* = 195	Cognition; brain structure/function (MRI); biomarkers (e.g. BDNF); gut microbiota: not reported	Cosupplementation did not improve cognitive outcomes or brain structure/function versus control. Microbiota not assessed/reported in main outcomes.
Nagpal et al., 2019	Modified Mediterranean–ketogenic diet (**MMKD**)	MMKD vs American Heart Association Diet (AHAD)	Randomized, double-blind, crossover; 6 weeks each arm; 6-week washout	*n* = 17 (CN = 6; MCI = 11)	Gut microbiome; fecal SCFAs; CSF AD biomarkers (A*β*40/42, total tau, p-tau181)	MMKD modulated gut microbiome and SCFAs and was associated with improved CSF AD biomarkers; taxa/SCFA–biomarker correlations differed by cognitive status
Nagpal et al., 2020	MMKD	MMKD vs AHAD	Single-center, randomized, double-blind **crossover**; 6 weeks each diet (pre/post)	*n* = 17 (MCI = 11; CN = 6)	Gut mycobiome (ITS1); correlations with CSF AD markers and gut bacteria	Identified MCI-specific mycobiome signatures; diets modulated mycobiome in association with AD markers, with MMKD showing broader effects in MCI
Dilmore et al., 2023	MMKD	MMKD vs AHAD	Randomized crossover; 6 weeks each diet; 6-week washout	Enrolled *n* = 23; completed *n* = 20	Shotgun metagenomics; metabolomics; foodomics; diet/cognition associations	After accounting for repeated measures, diet showed a larger effect on microbiome/metabolome than cognitive status; features differed by diet arm
Sakurai et al., 2022	Probiotic (heat-treated *Lactiplantibacillus plantarum* OLL2712)	Heat-treated OLL2712 cells powder (1 g/day; >5 × 10 ^ 9 cells) vs placebo powder (dextrin-based)	Randomized, double-blind, placebo-controlled; parallel groups; 12 weeks; single-site (Japan)	Randomized *n* = 81; analyzed *n* = 78 (≥65 years; declining memory; screening MPI <60)	Cognition (Cognitrax; composite memory, visual memory); dietary intake (BDHQ); gut microbiota (fecal 16S rDNA sequencing)	Adjusted analyses showed significant improvement in *composite memory* and *visual memory* in the active group; microbiota shifts included lower abundance ratio of inflammation-associated genera (*Lachnoclostridium, Monoglobus, Oscillibacter*).
Aljumaah et al., 2022	Probiotic (LGG)	*Lactobacillus rhamnosus* GG vs placebo	Randomized, double-blind, placebo-controlled; 3 months	*n* = 169	Cognitive measures and gut microbiota changes (trial outcomes reported pre/post)	Reported probiotic-associated changes in gut microbiota and cognitive-related measures (study-level outcomes summarized by arm)
Fei et al., 2023	Probiotic supplementation	Probiotic powder 2 g/day vs placebo	Randomized, double-blind, placebo-controlled; 12 weeks	*n* = 42 (21 probiotic; 21 placebo)	Cognitive function (MMSE, MoCA, ERP-P300), sleep quality (PSQI), anxiety/depression scales	Improvements were reported in cognitive and sleep-related measures in the probiotic group over the intervention period
McLeod et al., 2023	Mediterranean diet	Mediterranean diet ad libitum (MedA) vs Mediterranean diet with weight loss (MedWL)	8-month intervention; parallel groups	Total *n* = 66 (MedA = 31; MedWL = 35)	Gut microbiota composition/diversity; diet-related microbial functions; cognitive performance	Reported significant between-group differences in alpha diversity and time trends; explored microbiome features linked to cognitive performance
Ghosh et al., 2020 (NU-AGE)	Mediterranean diet (NU-AGE MedDiet)	NU-AGE MedDiet vs control (habitual diet / no tailored MedDiet program)	1-year, randomized, multicentre, single-blind, controlled trial	Pre/post gut microbiome profiling across five European countries *n* = 612 with paired microbiome profiles (controls = 289; NU-AGE MedDiet = 323) Gut microbiota (16S rRNA profiles); dietary adherence; frailty indices	Cognitive function measures; inflammatory markers MedDiet adherence was linked to specific microbiome shifts	Diet-enriched taxa correlated with lower frailty and inflammation markers and with better cognitive measures; inferred metabolite profiles suggested higher SCFA potential and lower production of secondary bile acids and other detrimental metabolites
**Observational studies (dietary exposure/assessment)**						
Zhang et al., 2021	Diet quality / inflammatory potential	CDGI-2018 (higher vs lower) and E-DII (lower vs higher); FFQ-based	Prospective cohort; baseline diet + cognitive tests; MCI at last follow-up; microbiota substudy	Cohort *n* = 2,239; microbiota/miRNA subgroup *n* = 127 (MCI = 75; controls = 52)	Primary: MCI diagnosis (MoCA + neurologist); fecal 16S (V4; QIIME)	Diet quality scores were associated with MCI risk; MCI vs controls showed gut microbiota composition differences (16S), and integrated models combined diet, microbiota and miRNAs
González-Domínguez et al., 2021	Nutrimetabolomics (diet-related + microbiota-derived metabolites)	Serum panel of food-related metabolites and microbiota-transformed derivatives	Nested case-control within 3C cohort; cognitive follow-up every 2–3 years; 12-year cognitive decline slopes	Bordeaux: 209 cases + 209 controls; Dijon: 212 + 212 (total *n* = 842)	Cognitive decline defined by composite Z-score across five neuropsych tests; targeted metabolomics including microbiota derivatives	Identified metabolite signatures linked to subsequent cognitive decline; several phenolic/metabolite patterns consistent with dietary sources and microbial metabolism showed inverse associations, while other metabolite patterns were associated with higher odds of decline

**Fig. 2:**
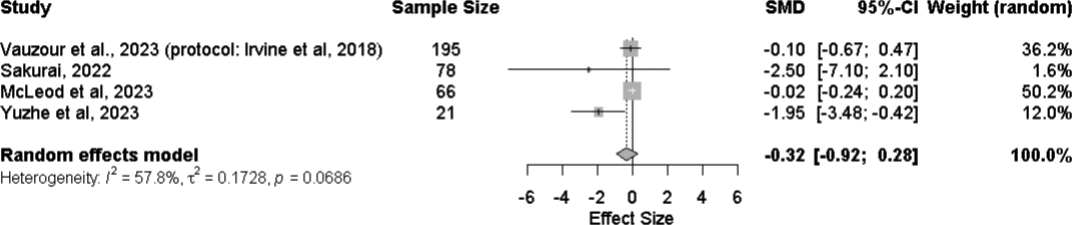
Forest plot of MCI.

CN: cognitively normal; MCI: mild cognitive impairment; MMKD: modified Mediterranean–ketogenic diet; AHAD: American Heart Association Diet; MedA: Mediterranean diet ad libitum; MedWL: Mediterranean diet with weight loss; SCFAs: short-chain fatty acids; CSF: cerebrospinal fluid; AD: Alzheimer’s disease; MMSE: Mini-Mental State Examination; MoCA: Montreal Cognitive Assessment; PSQI: Pittsburgh Sleep Quality Index; ERP-P300: event-related potential P300; MPI: Memory Performance Index; BDHQ: Brief-type self-administered Diet History Questionnaire; QIME: Quantitative Insights Into Microbial Ecology; rRNA: Ribosomal Ribonucleic Acid. Sample size refers to participants analyzed for the main outcomes (e.g. paired pre/post microbiome profiles) when reported. Main findings are qualitative summaries of study-level results (see Fig. 2 for pooled effect estimate where applicable).

The included studies reporting taxa-level differences indicate that gut microbial composition varies between CN adults and those with MCI. For instance, CN participants generally showed higher representation of taxa commonly linked to gut ecosystem stability and SCFA production (e.g. *Bifidobacterium* and *Faecalibacterium prausnitzii*), and several studies also noted a relative enrichment of *Dialister*. Conversely, MCI cohorts more often exhibited higher relative abundance of taxa frequently reported in dysbiotic or pro-inflammatory profiles, including *Enterobacteriaceae* and selected *Ruminococcus/Prevotella* taxa, with increases in *Ruminococcus* spp. and *Prevotella ruminicola* described more consistently across studies. However, the specific taxa identified and the direction of change varied across cohorts, sequencing/processing pipelines, and statistical approaches (Table 5).

**Table 5:** Comparison of gut microbiota composition in individuals with normal cognition and mild cognitive impairment

Authors / study context	Cognitively normal / controls (enriched / higher)	Mild cognitive impairment (enriched / higher)
Dilmore et al., 2023 (randomized crossover; shotgun metagenomics; negative binomial mixed-effects modeling)	*n* = 11 *Bacteroides fragilis* *Dialister invisus* *Cryptobacterium sp.* *Acinetobacter sp.*	*n* = 9 *Ruminococcus sp. CAG:330* *Higher Ruminococcus/Dialister ratio reported* *Sutterella wadsworthensis* *Enterobacter aerogenes*
Aljumaah (Mashael) et al., 2022 (RCT; baseline cross-sectional comparison by cognitive status; 16S rRNA amplicon + shotgun/WGS; qPCR; association analyses)	*n* = 125 *Bifidobacterium longum* *Bifidobacterium breve* *Faecalibacterium prausnitzii* *Sutterella faecalis* *Ruthenibacterium lactatiformans*	*n* = 44 *Prevotella ruminicola* *Bacteroides thetaiotaomicron* *Bacteroides xylanisolvens* *Citrobacter portucalensis*
Nagpal et al., 2020 (randomized crossover; fungal ITS1 profiling; LEfSe and network analyses)	*n* = 6 *Dialister*	*n* = 11 *Proteobacteria* *Enterobacteriaceae* *Coriobacteriaceae* *Phascolarctobacterium*
Zhang et al., 2021 (cross-sectional; fecal microbiota 16S V4; QIIME; LEfSe)	*n* = 52 *Bifidobacteriaceae*	*n* = 75 *Enterobacteriaceae* *Rhizobiaceae* *Megasphaera*
Nagpal et al., 2019 (randomized crossover; fecal 16S rRNA sequencing; group comparisons of differential taxa)	*n* = 6 Rumininococcaceae Lachnospiraceae Akkermansiaceae	*n* = 11 Veillonellaceae Enterobacteriaceae Bacteroidaceae

CAG: Co-Abundance Group; CN: cognitively normal; LEfSe: Linear Discriminant Analysis Effect Size; MCI: mild cognitive impairment; QIME: Quantitative Insights Into Microbial Ecology; qPCR: Quantitative Polymerase Chain Reaction; RCT: Randomized Controlled Trial; rRNA: Ribosomal Ribonucleic Acid; WGS: Whole-Genome Sequencing. Taxa are listed as reported by each source study for CN/control vs MCI (or cognitively intact vs cognitively impaired), alongside study-level context (sample size, assay, and analysis approach). Taxonomic rank varies across studies, and directionality depends on each study’s analytic pipeline and statistical model; the table summarizes qualitative directionality and does not imply causality.

The meta-analysis pooled data from 360 participants across four clinical trials to evaluate the effect of dietary interventions on cognitive decline. Given the expected variability between studies, a random-effects model was applied to provide an appropriate combined estimate and to ensure a suitable interpretation of the pooled effect. The pooled SMD under the random-effects model was −0.32 (95% CI: −0.92 to 0.28), indicating a mild trend favoring dietary interventions but without statistical significance. Moderate heterogeneity was observed (*I*^2^ = 57.8%, *p* = 0.0686), potentially reflecting differences in participant characteristics, the type and duration of dietary interventions, and the cognitive outcome measures used across trials, as well as differences in analytical approaches across studies. The corresponding forest plot is shown in Fig. 2.

Forest plot of the effect of dietary interventions on cognitive decline in adults with MCI) across four clinical trials (total *N* = 360). Effect estimates are SMDs with 95% confidence intervals, oriented so that negative values favor the dietary intervention (i.e. less cognitive decline / better cognition) versus the comparator. Squares represent individual study estimates (size proportional to the inverse-variance weight under the random-effects model), and horizontal lines indicate 95% CIs. The pooled random-effects estimate (DerSimonian–Laird; inverse-variance) is shown by the diamond; *I*^2^ and *τ*^2^ quantify between-study heterogeneity and the Cochran Q test *p*-value is reported below the plot. Abbreviations: CI: confidence interval; *I*^2^: inconsistency; *τ*^2^: between-study variance; SMD: standardized mean difference; MCI: mild cognitive impairment.

## Discussion

The relationship between diet, gut microbiota, and MCI has garnered increasing interest over the past decade, given the implications of the gut–brain axis in neurodegeneration (20, 24,25, 26). This systematic review/meta-analysis examines how certain dietary patterns can influence the gut microbiota and, in turn, cognitive health in individuals with MCI. Although the study’s findings provide an interesting overview, the dietary impact on cognition still presents controversies and significant methodological challenges.

The reviewed studies suggest that microbial composition varies considerably between individuals with MCI and those with normal cognition. For instance, the prevalence of beneficial bacteria such as *Bifidobacterium* and *Faecalibacterium prausnitzii* in cognitively healthy individuals is consistent with previous studies highlighting their anti-inflammatory role in modulating the gut–brain axis (4). Conversely, the predominance of pro-inflammatory bacteria such as *Ruminococcus* and *Enterobacteriaceae* in individuals with MCI supports the hypothesis that intestinal and systemic inflammation may contribute to neuroinflammation, a key mechanism in cognitive decline (7).

From a neurobiological perspective, the relationship between microbiota and cognition is mediated by several mechanisms, including the production of SCFAs and the modulation of neurotransmitters such as Gamma-Aminobutyric Acid and serotonin (5). These metabolites play a crucial role in regulating inflammation and neuroplasticity, which may explain why certain dietary patterns, like the Mediterranean diet, have neuroprotective effects (6). In contrast, the Western diet is associated with a reduction in microbial diversity and an increase in intestinal permeability, facilitating the entry of endotoxins into the bloodstream and activating inflammatory pathways that may negatively affect the brain (14).

Although the meta-analysis suggests a mild trend favoring dietary interventions for reducing cognitive decline, the confidence interval crosses the null value and heterogeneity is moderate. Differences in participant characteristics, intervention type and duration, and the cognitive outcome measures used may have contributed to variability across trials. However, the descriptive evidence across studies remains compatible with the possibility that dietary patterns associated with greater microbial diversity (e.g. higher fiber and polyphenol intake) could support cognitive health over longer follow-up periods (18).

An aspect that deserves more attention in future research is the identification of specific microbiota biomarkers that may correlate more directly with cognitive outcomes. Although this study identified differences in specific bacterial genera between individuals with and without MCI, integrating more detailed analyses of microbial metabolites and their impact on brain neurochemistry could provide a deeper understanding of the mechanisms involved.

## Strengths and limitations

This review offers a distinctive strength: it isolates the MCI phenotype and quantitatively synthesizes diet-cognition associations while providing a parallel, taxa-level synopsis of gut microbiota signals reported alongside dietary exposures, addressing recognized reporting gaps in human microbiome studies and facilitating comparability across studies (27). In addition, heterogeneous cognitive scales were harmonized using SMDs under random-effects models with an explicit interpretation of heterogeneity, enabling cautious, like-for-like estimation across instruments (28). Notwithstanding these strengths, several limitations remain. Among these, a key limitation is the lack of detailed analyses on microbial metabolites and neurochemical impacts, largely due to insufficient data in the reviewed articles. Additionally, significant variability in the type, duration, and frequency of dietary interventions complicates direct comparisons and consistent pattern identification. Shorter intervention durations may not allow sufficient time to observe meaningful changes in microbiota composition or cognition. Furthermore, the non-standardized tools and methods used to assess cognitive decline introduce variability, limiting the comparability of intervention outcomes.

## Conclusion

This systematic review/meta-analysis underscores the importance of diet in modulating gut microbiota and its potential impact on cognition in individuals with MCI. Although the observed effects are not statistically significant, the direction of the findings supports the hypothesis that dietary intervention can influence cognitive health through microbiome modulation. Future research should focus on improving the standardization of dietary interventions and outcome measures, as well as integrating metabolite analyses to clarify the exact mechanisms linking gut microbiota with cognitive decline.
